# Altered neuromuscular control mechanisms of the trapezius muscle in fibromyalgia

**DOI:** 10.1186/1471-2474-11-42

**Published:** 2010-03-05

**Authors:** Björn Gerdle, Christer Grönlund, Stefan J Karlsson, Andreas Holtermann, Karin Roeleveld

**Affiliations:** 1 Rehabilitation Medicine, Department of Clinical and Experimental Medicine, Linköping University, SE-581 85 Linköping, Sweden; 2 Pain and rehabilitation Centre, University Hospital, SE-581 85 Linköping, Sweden; 3 Biomedical Engineering & Informatics, University Hospital, SE-981 85 Umeå, Sweden; 4 Centre for Biomedical Engineering and Physics, Umeå University, Umeå, Sweden; 5 National Research Centre for the Working Environment, Lersø Parkallé 105, DK-2100 Copenhagen, Denmark; 6 Human Movement Science Programme, Dragvoll, Norwegian University of Science and Technology, N-7491 Trondheim, Norway

## Abstract

**Background:**

fibromyalgia is a relatively common condition with widespread pain and pressure allodynia, but unknown aetiology. For decades, the association between motor control strategies and chronic pain has been a topic for debate. One long held functional neuromuscular control mechanism is differential activation between regions within a single muscle. The aim of this study was to investigate differences in neuromuscular control, i.e. differential activation, between myalgic trapezius in fibromyalgia patients and healthy controls.

**Methods:**

27 fibromyalgia patients and 30 healthy controls performed 3 minutes bilateral shoulder elevations with different loads (0-4 Kg) with a high-density surface electromyographical (EMG) grid placed above the upper trapezius. Differential activation was quantified by the power spectral median frequency of the difference in EMG amplitude between the cranial and caudal parts of the upper trapezius. The average duration of the differential activation was described by the inverse of the median frequency of the differential activations.

**Results:**

the median frequency of the differential activations was significantly lower, and the average duration of the differential activations significantly longer in fibromyalgia compared with controls at the two lowest load levels (0-1 Kg) (p < 0.04), but not at the two highest load levels (2 and 4 Kg).

**Conclusion:**

these findings illustrate a different neuromuscular control between fibromyalgia patients and healthy controls during a low load functional task, either sustaining or resulting from the chronic painful condition. The findings may have clinical relevance for rehabilitation strategies for fibromyalgia.

## Background

Approximately 20% of the European population report severe chronic musculoskeletal pain [1]. Ten to twenty percent of those, that is 2-4 percent of the general population, suffer from fibromyalgia (FM). FM is characterized by a widespread pain and tenderness to pressure (allodynia), but with unknown aetiology [2-4]. FM has been described as a complex hyperalgesic pain syndrome, in which abnormalities of central sensory processing interact with peripheral pain generators and psycho-neuro-endocrine dysfunction [5-7].

FM patients often report pain and tenderness in the trapezius muscle, reflected by the numerous predefined tender points located within the muscle [8]. Moreover, the trapezius muscle in FM has shown mitochondrial disturbances in type-I muscle fibers (i.e., ragged-red fibers and moth-eaten fibers), hypotrophy of type-II fibers, reduced capillarisation [9] and altered microcirculation [10,11].

In line with the disturbed microcirculation in muscles of FM patients, elevated levels of muscle fatigue has been reported in patients with chronic neck pain [12]. Reports of inability to relax muscle or increased muscular tension in persons with or developing trapezius myalgia [13-17] have brought focus on the association between neuromuscular control mechanisms and chronic pain. Several neuromuscular control mechanisms are proposed to prevent continuing muscle activity, local circulation disturbances, fatigue, high-threshold afferent activity and pain [18]. An often discussed neuromuscular control mechanism is rotation or substitution of active single motor units [19,20], assumed to prevent fatigue during prolonged contractions. Another related neuromuscular control mechanism suggested to prevent monotonous prolonged activation of motor units and local fatigue is reciprocal reversals of activity between regions within a single muscle, termed differential activation [21-23].

Recently, Falla and colleagues observed a relatively higher increase in activity of the cranial compared to the caudal part of the upper trapezius in healthy controls compared to FM patients during a sustained contraction [24]. This finding indicates recruitment of more motor units in the cranial part of the upper trapzius with fatigue in FM patients compared to healthy controls [24]. However, it does not provide information whether patients with FM possess an altered neuromuscular control strategy involving shifts in activity between regions within a muscle (i.e. differential activations).

The aim of this study was to examine whether neuromuscular control is different in patients with FM from healthy controls with respect to differential activation within the trapezius muscle during shoulder elevations with low load. Such low load elevations are functional tasks which can reveal altered neuromuscular control mechanisms relevant for daily living activities and are therefore of ecologic validity.

## Methods

### Subjects

#### Healthy controls

Female healthy controls (N = 30; age: 40 ± 5 years, weight: 62.8 ± 7.3 kg, height: 168 ± 8 cm) without ongoing acute or chronic pain were recruited through advertisements among students and staff at the University Hospital of Linköping, Sweden. None of the subjects in the control group had pain according to a rating of pain intensity in the neck and shoulder region using a 100 mm visual analogue scale (VAS; endpoints: no pain and maximum pain, respectively) or had a recurrent and/or chronic pain condition.

#### Fibromyalgia patients (FM)

Female patients with fibromyalgia (N = 29; age: 37 ± 5 years, weight: 69.3 ± 9.5 kg, height: 166 ± 5 cm), were recruited from the Pain and Rehabilitation Centre, University Hospital, Linköping, Sweden. It should be noted that not all of these patients were able to perform all tasks (see Results, Drop-out and activity levels at the different loads). The patients were offered participation in the study after examination of medical journals, positive response to information letter, and a phone conversation with a physician. The patients were clinically diagnosed according to the ACR criteria of 1990 for the classification of fibromyalgia (FM) [25]. The mean duration of FM was 6.6 ± 3.2 years (minimum 2 years), and the maximum pain intensity recent week was 87 ± 13 mm according to a 100 mm VAS. Corresponding ratings for minimum pain intensity recent week, and average pain intensity recent week were 25 ± 15 mm, and 50 ± 18 mm, respectively.

Basic descriptive data of the two groups of subjects (i.e., age anthropometric data, habitual pain intensity, and data from ultrasound recording) have been presented previously [26].

#### Ethical aspects

All subjects gave written informed consent to participate. The study conformed to the Declaration of Helsinki and the study protocol was approved by the local ethics committee at Linköping University.

### Ultrasound recordings

Ultrasound measurements were taken of the thickness (mm) of the trapezius muscle and the subcutaneous soft tissue (skin and fat tissue taken together) 2 cm lateral of the midpoint between the seventh process of the cervical spine and the lateral part of the acromion process using an Acuson 128XP/10 (Siemens).

### Muscle contractions

**Figure 1:**
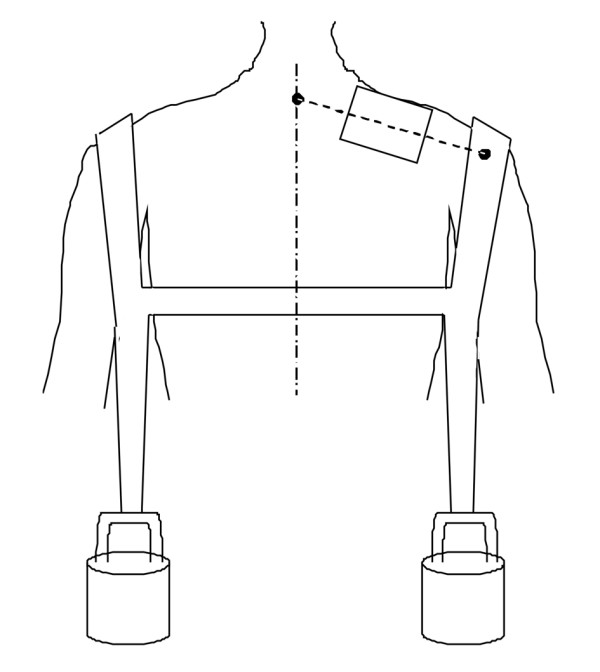
**Experimental setup**. Posterior view of a subject, electromyographical electrode device (rectangle), and weight harness. The centre of the electrode-device was placed in the middle of the line between processus spinosus of the C7 vertebra and the lateral edge of acromion. The harness was used to attach weights symmetrically on the shoulders. Subjects performed isometric shoulder elevation such that their shoulders were held in the horizontal plane.

The subjects performed symmetrical bilateral shoulder elevations with different loads. The loads were applied through the attachment of different weights on a harness (Figure 1). The harness consisted of 2 belts hanging on both shoulders on the level of the acromion to allow attachment of the weights. One additional belt was placed around the torso in order to fixate the other two belts. After attaching the harness and weights, the subjects were asked to lift the weights such that the shoulders were in a horizontal plane, and to hold this position for 3 min. Weights of 0, 1, 2, and 4 kg were applied successively. The subjects were given 1-min rest between each contraction. The choice of absolute weights and not relative weights in relation to maximum performance was based on the difficulties for obtaining valid maximum voluntary contractions (MVC) in FM patients due to pain and/or psychological aspects such as fear-avoidance or kinesiophobia.

### Surface EMG acquisition

Surface electromyographic (EMG) signals were recorded using a high-density electrode-grid (modified ActiveOne, BioSemi, Amsterdam, Netherlands) consisting of 13 by 10 active electrodes, covering 6 × 4.5 cm of the skin surface. The base of the electrode-grid device was concave and semiflexible and thereby fitted well with the convex recordings area of the upper trapezius muscle. The centre of the electrode grid was placed on the skin above the right trapezius muscle in the middle of the line between the processus spinosus of the seventh cervical vertebra and the lateral edge of acromion (Figure 1). In this way, the recorded signals of all subjects were not affected by muscle-tendon transitions and similarly affected by the motor end plate region. To generate a stable pressure between electrodes and the skin, the electrode grid was held in place by two elastic straps around the shoulder and torso of the subject. The surface EMG signals were recorded from all electrodes with a common reference on the processus spinosus of the seventh cervical vertebra at 2048 Hz.

### Surface EMG analysis - Quantification of differential activation

The differential activation analysis was previously described [23]. The essence of the analysis was to examine the temporal changes of the difference in myoelectric activity between two regions within a muscle. While the EMG signals were recorded using a 2-D high-density EMG grid, a large part of the processing (most of step II below) was undertaken in order to reduce the data to signals originating from the caudal and cranial regions of the trapezius muscle. Multiple channels covering a large area were recorded to get a better estimate of the activation of the two muscle parts [27]. In short, the procedure involved;

I. Pre-processing: Prior to analysis, poor quality signals were omitted. The remaining signals were high-pass filtered at 10 Hz, and bipolar spatial filtering in the fiber direction (medial-lateral) was carried out.

**Figure 2:**
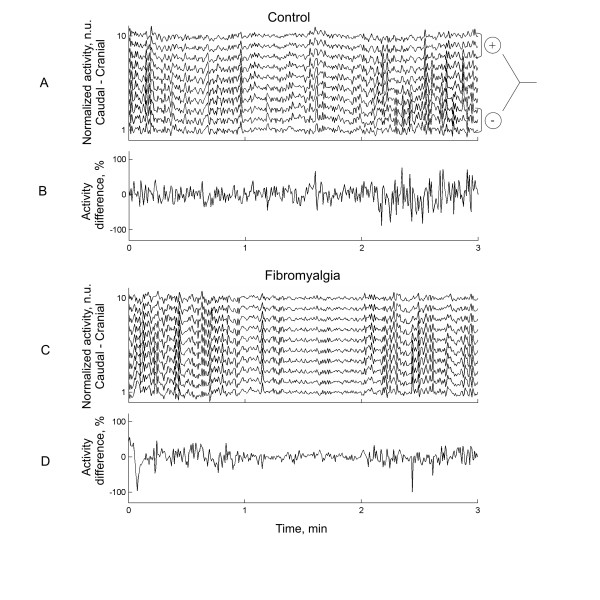
**Examples of a normalized muscle activity from a high-density EMG recording (caudal - cranial, channel 1 to 10, 5 mm's apart) from one healthy control (A) and one patient with fibromyalgia (C) performing a 1 kg weight of isometric shoulder elevation**. The activity difference (B and D) in normalized activity between caudal and cranial regions segments (see A right) were used to calculate the power spectral median frequency.

II. Muscle activity in the cranial - caudal direction: Muscle activity in the caudal - cranial direction (perpendicular to fibre orientation) was calculated by averaging the muscle activity recorded by electrodes of the grid along the fibre orientation (medial - lateral), providing 10 activity signals in the cranial caudal direction. The muscle activity level of the EMG signals was described using the root-mean-square (RMS), calculated in 0.5 s non-overlapping time-windows. In order to compare the activity between regions within the muscle, the muscle activity signals were de-trended and normalized to their maximum (Figure 2A and 2C).

III. Activity difference: The difference in the normalized muscle activity between the cranial (average of the three most superior electrode positions) and the caudal (average of the three most inferior electrode positions) regions of the upper trapezius was calculated (see Figure 2A).

IV. Median frequency and average duration of the differential activations: Differential activation was quantified using the power spectral median frequency of the activity difference signal. The inverse of the median frequency of the differential activations was calculated to quantify the average duration of the differential activations.

### Statistics

All statistical analyses were performed using MATLAB (The Mathworks, Nattick, USA; version 2007b). For variables and indices mean value with one standard deviation (± 1 SD) are generally reported. For multiple comparisons between the two groups, two-way analysis of variance (ANOVA) was performed. Pair-wise comparisons were performed using Student's t-test. A probability of ≤ 0.05 (two-tailed) was used as criteria for significance in all tests.

## Results

### Anthropometric data including muscle dimensions (also reported in [26])

FM patients were significantly younger (p = 0.017) and heavier (p = 0.005) than control subjects. The average thickness of the trapezius muscle was similar in FM and controls, being 11.7 ± 2.4 mm over the examination area of the trapezius in FM and 11.6 ± 2.4 mm in CON (p = 0.937). The thickness of the superjacent tissues (skin and subcutaneous tissue) was somewhat thicker in FM than in controls (6.6 ± 1.6 mm over the examination area in FM versus 5.2 ± 2.5 mm in controls; p = 0.028).

### Drop-outs and activity levels at the different load levels

All control subjects (N = 30) were able to perform the contractions at the four load levels (0, 1, 2 and 4 kg) with a duration of 3 min, and were thus included in the subsequent analyses. In the FM group (N = 29), 2 (6.9%), 2 (6.9%), 4 (13.8%), and 8 (27.6%) patients were not able to perform the tasks during 3 min with the 4 different external loads, respectively, and were therefore excluded in the subsequent analyses for these load levels.

For all loads, the muscle activity levels averaged over all bipolar electrode leads was lower in FM than in controls (p < 0.001). More specific, the activity levels at the 4 different load levels (0, 1, 2 and 4 kg) in controls was respectively 0.87 ± 0.37, 1.04 ± 0.50, 1.14 ± 0.51 and 1.38 ± 0.58 mV and in FM 0.54 ± 0.24, 0.57 ± 0.32, 0.68 ± 0.33 and 0.82 ± 0.41 mV.

### Prevalence of differential activation

**Figure 3:**
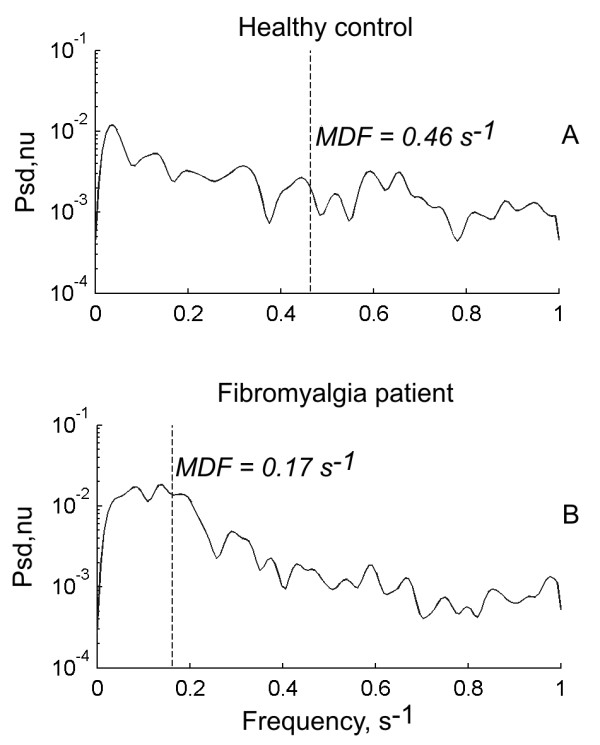
**Examples of the power spectral density (Psd) of the differential activity signal between the cranial and caudal parts of the trapezius and its corresponding median frequency (MDF), for a healthy control (A) and a patient with fibromyalgia (B)**.

Figure 2 illustrates a typical example of differential muscle activation between the cranial and caudal parts of the upper trapezius muscle with an external loading of 2 Kg from a control subject and a FM patient. As shown in the figure, the frequency of differential activity between the cranial and caudal parts of the trapezius was higher in the control subject (Figure 2A and 2B) compared to the FM patient (Figure 2C and 2D). Accordingly, the median frequency of the differential activation between the cranial and caudal regions of the trapezius was higher in a typical control subject (Figure 3A) than in a typical FM patient (Figure 3B) with an external loading of 2 Kg.

### Frequency of differential activation

**Figure 4:**
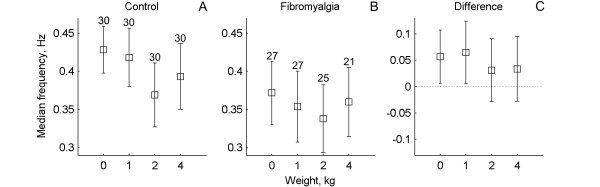
**Mean values and 95% confidence intervals for median frequency of the differential activations for healthy controls and patients with fibromyalgia at different weights**. Furthest to the right is shown the difference between the two groups.

The median frequency of differential activation at the two lowest load levels at the shoulders (i.e., 0 and 1 kg) was significantly lower in FM compared with the healthy controls (p < 0.04, Table 1, Figure 4). However, the frequency of differential activation was not significantly different between controls and FM at the two higher load levels (i.e., 2 and 4 Kg) (p > 0.28, Table 1, Figure 4). The median frequency of differential activation decreased with increasing loading of the neck-shoulder region in controls, but was invariant of the external loading in FM (Table 1).

**Table 1:** Median frequency and duration of differential activation in fibromyalgia (FM) and healthy controls (CON); mean values ± 1 standard deviation (SD) and minimum and maximum values.

*Group*	CON		FM		Statistics
*Variable and load*	Mean ± SD	Min-Max	Mean ± SD	Min-Max	p-value
***Frequency *(*s*^-1^)**					
**0 kg**	0.43 ± 0.08	0.24-0.56	0.37 ± 0.11	0.14-0.53	0.028*
**1 kg**	0.42 ± 0.10	0.23-0.57	0.35 ± 0.12	0.12-0.60	0.033*
**2 kg**	0.37 ± 0.11	0.11-0.51	0.34 ± 0.12	0.14-0.53	0.303
**4 kg**	0.39 ± 0.11	0.14-0.56	0.36 ± 0.12	0.14-0.60	0.280
***Duration (sec)***					
**0 kg**	1.21 ± 0.26	0.89-2.08	1.51 ± 0.61	0.95-3.59	0.022*
**1 kg**	1.28 ± 0.36	0.88-2.23	1.67 ± 0.85	0.82-4.30	0.025*
**2 kg**	1.57 ± 0.83	0.98-4.60	1.71 ± 0.73	0.95-3.59	0.500
**4 kg**	1.42 ± 0.59	0.90-3.59	1.59 ± 0.68	0.84-3.58	0.325

Furthest to the right is the result of the statistical comparisons (p-values) between groups (CON vs. FM); * denotes significant difference.

### Duration of differential activation

The average duration of differential activation at the two lowest load levels at the shoulders (i.e., 0 and 1 kg) was significantly lower in FM compared with the healthy controls (p < 0.03, Table 1). However, the average duration of differential activation was not significantly different between controls and FM at the two higher load levels (i.e., 2 and 4 Kg) (p > 0.32, Table 1). The average duration of differential activation did not vary with modifying external loading of the neck-shoulder region in neither controls nor FM (Table 1).

## Discussion

The main finding of this study was the lower median frequency of the differential activations, and thus a longer average duration of differential activation between the trapezius regions in fibromyalgia patients compared with healthy controls during static shoulder elevation with none or very low weights. These results will be discussed in the light of mechanisms behind trapezius myalgia in general, and FM in specific.

The neuromuscular control of patients with musculoskeletal pain has received clinical interest for more than a century [28]. For example based on clinical observations that patients with myalgia have tender muscles, it has often been assumed that a vicious circle of pain and hyperactivity exists in chronic pain [29]. Because the healthy controls seem to be able to utilize a redundancy of motor unit populations more frequently through shifting active motor unit populations than patients with fibromyalgia, this finding fits with the Cinderella hypothesis [30].

Already in 1922, Forbes [31] noted that subpopulations of motor units within a muscle may shift on being active (differential activation) and therefore rotate on the loading of the muscle fibers. Shifts in activity between motor unit subpopulations in anatomical complex muscles during monotonous contractions are therefore considered to prevent local muscle fatigue and muscle fiber overexertion [32]. Accordingly, subjects with larger spatial changes in distribution of intra-muscular activity within the upper trapezius are observed to be able to sustain a static contraction for longer duration than subjects with minor spatial changes [33]. The ability to shift activity between regions within the upper trapezius may be a preventive mechanism for avoiding reflexive mediated muscle hyperactivity from acute painful condition further enhancing pain and a chronic condition [34]. Therefore, a longer duration of differential activation of activity within the upper trapezius may be an underlying neuromuscular control mechanism associated with increased risk for acute and chronic musculoskeletal symptoms. In a previous study [23], the duration of differential activation was observed to be positively associated with fatigue prevention. However, this was observed at the biceps brachii during a 30 min contraction in healthy individuals, and may therefore not be translated to this study of the trapezius muscle in FM and healthy controls during a 3 min contraction.

On the other hand, the reduced differential activation in fibromyalgia patients may also be a result of the chronic painful condition; e.g in CNS (central sensitizing processes, alterations in function of neuromatrix of pain in the brain or in descending inhibition) [35-38] and/or in muscle [9-11]. This is supported by the finding that myoelectrical manifestations of fatigue in fibromyalgia patients are central of origin [39]. Prospective investigations of the relation between neuromuscular control strategies and chronic pain are very few [17], and future prospective investigations between neuromuscular control strategies and chronic pain are recommended for elucidating the relation between neuromuscular control and chronic myalgia.

The lower EMG amplitudes in FM can eventually partly be due to the somewhat thicker subcutaneous tissue. Although a thicker subcutaneous layer can affect EMG amplitude and the spatial distribution, it is not likely to affect temporal fluctuations in EMG amplitude and differential activation as investigated in the present study. In addition, the ultrasound investigation did not reveal any significant difference in thickness of the trapezius muscle between the two groups. In order to definitely determine the level of muscle activity, an MVC is needed. However, the validity of MVC in FM can be questioned due to factors such as pain, increase in pain with increasing contraction level, anxiety and different aspects of fear and fear behaviour. However, the observation of a difference in differential activations between FM patients and health controls at very low force levels (0-1 Kg), but not higher force levels (2-4 Kg), supports that a generally higher maximal strength of controls compared with FM patients [24] is not the primary cause to the findings of this study.

The previously documented changes in intra-muscular activity within the upper trapezius of healthy subjects with injection of hypertonic saline suggest that local elicitation of nociceptive afferents generates a reorganization of activity within the upper trapezius [40]. The ability of independent selective voluntary activation of regions within the trapezius muscle with biofeedback guidance [41] illustrates that differential activation between regions of the upper trapezius may be a voluntary neuromuscular strategy.

## Conclusions

For the first time, a different neuromuscular control mechanism involving degree of shifts between regions (differential activation) within a single muscle is observed between fibromyalgia patients and healthy controls. The lower frequency of differential activation between regions within the upper trapezius of FM patients than controls indicates that this may be a neuromuscular control strategy either sustaining or being a result of the chronic painful condition. The findings may have clinical relevance for rehabilitation strategies for fibromyalgia.

## Competing interests

The authors declare that they have no competing interests.

## Authors' contributions

BG, CG, KR and SJK coordinated and performed the study. CG analyzed the data and made the statistical analyses. BG and AH wrote the first draft of the manuscript. All authors contributed to the conception, design, interpretation of data, and critically revising the manuscript. All authors approved the final manuscript.

## Pre-publication history

The pre-publication history for this paper can be accessed here:

http://www.biomedcentral.com/1471-2474/11/42/prepub

## References

[B1] Breivik H Collett B Ventafridda V Cohen R Gallacher D Survey of chronic pain in Europe: Prevalence, impact on daily life, and treatment European Journal of Pain 2006 10 287 333 10.1016/j.ejpain.2005.06.009 16095934

[B2] Lawrence RC Helmick CG Arnett FC Deyo RA Felson DT Giannini EH Heyse SP Hirsch R Hochberg MC Hunder GG Liang MH Pillemer SR Steen VD Wolfe F Estimates of the prevalence of arthritis and selected musculoskeletal disorders in the United States Arthritis and Rheumatism 1998 41 778 799 10.1002/1529-0131(199805)41:5<778::AID-ART4>3.0.CO;2-V 9588729

[B3] White KP Speechley M Harth M Ostbye T The London fibromyalgia epidemiology study: Comparing the demographic and clinical characteristics in 100 random community cases of fibromyalgia versus controls Journal of Rheumatology 1999 26 1577 1585 10405948

[B4] Wolfe F Ross K Anderson J Russell IJ Hebert L The prevalence and characteristics of fibromyalgia in the general-population Arthritis and Rheumatism 1995 38 19 28 10.1002/art.1780380104 7818567

[B5] Bennett RM Emerging concepts in the neurobiology of chronic pain: Evidence of abnormal sensory processing in fibromyalgia Mayo Clinic Proceedings 1999 74 385 398 10.4065/74.4.385 10221469

[B6] Staud R The role of peripheral input for chronic pain syndromes like fibromyalgia syndrome Journal of Musculoskeletal Pain 2008 16 67 74 10.1080/10582450801960339

[B7] Vierck CJJ Mechanisms underlying development of spatially distributed chronic pain (fibromyalgia) Pain 2006 124 242 263 10.1016/j.pain.2006.06.001 16842915

[B8] Mease P Fibromyalgia syndrome: Review of clinical presentation, pathogenesis, outcome measures, and treatment Journal of Rheumatology 2005 32 6 21 16078356

[B9] Bengtsson A The muscle in fibromyalgia Rheumatology 2002 41 721 724 10.1093/rheumatology/41.7.721 12096218

[B10] Elvin A Siosteen AK Nilsson A Kosek E Decreased muscle blood flow in fibromyalgia patients during standardised muscle exercise: A contrast media enhanced colour doppler study European Journal of Pain 2006 10 137 144 10.1016/j.ejpain.2005.02.001 16310717

[B11] Sandberg M Larsson B Lindberg LG Gerdle B Different patterns of blood flow response in the trapezius muscle following needle stimulation (acupuncture) between healthy subjects and patients with fibromyalgia and work-related trapezius myalgia European Journal of Pain 2005 9 497 510 10.1016/j.ejpain.2004.11.002 16139178

[B12] Falla D Farina D Muscle fiber conduction velocity of the upper trapezius muscle during dynamic contraction of the upper limb in patients with chronic neck pain Pain 2005 116 138 145 10.1016/j.pain.2005.03.038 15927379

[B13] Elert J Kendall SA Larsson B Mansson B Gerdle B Chronic pain and difficulty in relaxing postural muscles in patients with fibromyalgia and chronic whiplash associated disorders Journal of Rheumatology 2001 28 1361 1368 11409132

[B14] Elert JE Rantapää-Dahlqvist SB Henriksson-Larsen K Lorentzon R Gerdle BUC Muscle performance, electromyography and fiber type composition in fibromyalgia and work-related myalgia Scandinavian Journal of Rheumatology 1992 21 28 34 10.3109/03009749209095059 1570484

[B15] Elert JE Dahlqvist SBR Henrikssonlarsen K Gerdle B Increased EMG activity during short pauses in patients with primary fibromyalgia Scandinavian Journal of Rheumatology 1989 18 321 323 10.3109/03009748909095036 2595350

[B16] Hägg G Åström A Load pattern and pressure pain threshold in the upper trapezius muscle and psychosocial factors in medical secretaries with and without shoulder/neck disorders Int Arch Occup Environ Health 1997 69 423 432 10.1007/s004200050170 9215929

[B17] Veiersted KB Westgaard RH Andersen P Electromyographic evaluation of muscular work pattern as a predictor of trapezius myalgia Scand J Work Environ Health 1993 19 284 290 8235518 10.5271/sjweh.1472

[B18] Falla D Bilenkij G Jull G Patients with chronic neck pain demonstrate altered patterns of muscle activation during performance of a functional upper limb task Spine 2004 29 1436 1440 10.1097/01.BRS.0000128759.02487.BF 15223935

[B19] Bawa P Pang MY Olesen KA Calancie B Rotation of motoneurons during prolonged isometric contractions in humans Journal of Neurophysiology 2006 96 1135 1140 10.1152/jn.01063.2005 16775202

[B20] Westgaard RH De Luca CJ Motor unit substitution in long-duration contractions of the human trapezius muscle Journal of Neurophysiology 1999 82 501 504 10400978 10.1152/jn.1999.82.1.501

[B21] Holtermann A Grönlund C Karlsson JS Roeleveld K Differential activation of regions within the biceps brachii muscle during fatigue Acta Physiologica 2008 192 559 567 10.1111/j.1748-1716.2007.01775.x 18005216

[B22] Chanaud CM Pratt CA Loeb GE Functionally complex muscles of the cat hindlimb. V. The roles of histochemical fiber-type regionalization and mechanical heterogeneity in differential muscle activation Experimental Brain Research 1991 85 300 313 10.1007/BF00229408 1832646

[B23] Holtermann A Grönlund C Ingebrigtsen J Karlsson JS Roeleveld K Duration of differential activations is functionally related to fatigue prevention during low-level contractions Journal of Electromyography and Kinesiology 2010 20 241 245 10.1016/j.jelekin.2009.04.011 19481957

[B24] Falla D Andersen H Danneskiold-Samsøe B Arendt-Nielsen L Farina D Adaptations of upper trapezius muscle activity during sustained contractions in women with fibromyalgia Journal of Electromyography and Kinesiology 2009 in press 10.1016/j.jelekin.2009.07.002 19682926

[B25] Wolfe F Smythe HA Yunus MB Bennett RM Bombardier C Goldenberg DL Tugwell P Campbell SM Abeles M Clark P Fam AG Farber SJ Fiechtner JJ Franklin CM Gatter RA Hamaty D Lessard J Lichtbroun AS Masi AT Mccain GA Reynolds WJ Romano TJ Russell IJ Sheon RP The American college of rheumatology 1990 Criteria for the classification of fibromyalgia - Report of the multicenter criteria committee Arthritis and Rheumatism 1990 33 160 172 10.1002/art.1780330203 2306288

[B26] Gerdle B Ostlund N Gronlund C Roeleveld K Karlsson JS Firing rate and conduction velocity of single motor units in the trapezius muscle in fibromyalgia patients and healthy controls Journal of Electromyography and Kinesiology 2008 18 707 716 10.1016/j.jelekin.2007.02.016 17459728

[B27] Staudenmann D Kingma I Stegeman DF van Dieen JH Towards optimal multi-channel EMG electrode configurations in muscle force estimation: a high density EMG study Journal of Electromyography and Kinesiology 2005 15 1 11 10.1016/j.jelekin.2004.06.008 15642649

[B28] Hough T Ergographic studies in muscular soreness American Journal of Physiology 1902 7 76 92

[B29] Travell J Rinzler S Herman M Pain and disability of the shoulder and arm - Treatment by intramuscular infiltration with procaine hydrochloride Journal of the American Medical Association 1942 120 417 422

[B30] Hägg G Anderson PA, Hobart DJ, Danoff JV Static work loads and occupational myalgia - a new explanation model Electromyographical kinesiology 1991 Amsterdam: Elsevier Science Publishers 141 144

[B31] Forbes A The interpretation of spinal reflexes in terms of present knowledge of nerve conduction Physiological Reviews 1922 2 361 414

[B32] Mathiassen SE The influence of exercise/rest schedule on the physiological and psychophysical response to isometric shoulder-neck exercise European Journal of Applied Physiology 1993 67 528 539 10.1007/BF00241650 8149933

[B33] Farina D Leclerc F Arendt-Nielsen L Buttelli O Madeleine P The change in spatial distribution of upper trapezius muscle activity is correlated to contraction duration Journal of Electromyography and Kinesiology 2008 18 16 25 10.1016/j.jelekin.2006.08.005 17049273

[B34] Johansson H Sojka P Pathophysiological mechanisms involved in genesis and spread of muscular tension in occupational muscle pain and in chronic musculoskeletal pain syndromes: A hypothesis Medical Hypotheses 1991 35 196 203 10.1016/0306-9877(91)90233-O 1943863

[B35] Banic B Petersen-Felix S Andersen OK Radanov BP Villiger PM Arendt-Nielsen L Curatolo M Evidence for spinal cord hypersensitivity in chronic pain after whiplash injury and in fibromyalgia Pain 2004 107 7 15 10.1016/j.pain.2003.05.001 14715383

[B36] Giesecke T Gracely RH Grant MAB Nachemson A Petzke F Williams DA Clauw DJ Evidence of augmented central pain processing in idiopathic chronic low back pain Arthritis and Rheumatism 2004 50 613 623 10.1002/art.20063 14872506

[B37] Gracely RH Petzke F Wolf JM Clauw DJ Functional magnetic resonance imaging evidence of augmented pain processing in fibromyalgia Arthritis and Rheumatism 2002 46 1333 1343 10.1002/art.10225 12115241

[B38] Graven-Nielsen T Kendall SA Henriksson KG Bengtsson M Sorensen J Johnson A Gerdle B Arendt-Nielsen L Ketamine reduces muscle pain, temporal summation, and referred pain in fibromyalgia patients Pain 2000 85 483 491 10.1016/S0304-3959(99)00308-5 10781923

[B39] Casale R Sarzi-Puttini P Atzeni F Gazzoni M Buskila D Rainoldi A Central motor control failure in fibromyalgia: a surface electromyography study BMC Musculoskeletal Disorders 2009 10 78 10.1186/1471-2474-10-78 19570214 PMC2714295

[B40] Falla D Farina D Graven-Nielsen T Experimental muscle pain results in reorganization of coordination among trapezius muscle subdivisions during repetitive shoulder flexion Experimental Brain Research 2007 178 385 393 10.1007/s00221-006-0746-6 17051373

[B41] Holtermann A Roeleveld K Mork PJ Grönlund C Karlsson JS Andersen LL Olsen HB Zebis MK Sjøgaard G Søgaard K Selective activation of neuromuscular compartments within the human trapezius muscle Journal of Electromyography and Kinesiology 2009 19 896 902 10.1016/j.jelekin.2008.04.016 18585928

